# Development and Internal Validation of a Preoperative Risk Stratification Model for Difficult Laparoscopic Cholecystectomy in Acute Cholecystitis

**DOI:** 10.3390/diagnostics16193191

**Published:** 2026-10-01

**Authors:** Sinan Aslan, Şebnem Çimen

**Affiliations:** 1 Department of General Surgery, Mersin City Training and Research Hospital, 33240 Mersin, Türkiye; drsinanaslan84@gmail.com; 2 Department of General Surgery, Akyurt State Hospital, 06750 Ankara, Türkiye

**Keywords:** acute cholecystitis, laparoscopic cholecystectomy, difficult laparoscopic cholecystectomy, preoperative risk stratification, CALLY index

## Abstract

**Background:** Difficult laparoscopic cholecystectomy (DLC) in patients with acute cholecystitis (AC) is associated with greater operative complexity and increased perioperative morbidity, yet reliable preoperative risk stratification remains limited. This study aimed to develop and internally validate a preoperative prediction model for DLC and to compare its predictive performance with that of an alternative C-reactive protein–albumin–lymphocyte (CALLY)-based model. **Materials and Methods:** We retrospectively analyzed consecutive adult patients who underwent laparoscopic cholecystectomy (LC) for AC at a single center between 1 January 2022 and 31 December 2025. DLC was defined as operative time >90 min, conversion to open surgery, subtotal cholecystectomy or a Nassar grade ≥4. Preoperative clinical, laboratory and ultrasonographic variables were evaluated using multivariable logistic regression. Model performance was assessed by discrimination, calibration, Brier score, decision curve analysis and bootstrap internal validation. **Results:** Among 8530 screened patients, 7736 met the inclusion criteria and 1736 (22.4%) experienced DLC. In the primary multivariable model, Tokyo Guidelines 2018 (TG18) grade II, TG18 grade III and preoperative C-reactive protein (CRP) were independently associated with DLC. The model demonstrated modest discrimination (area under curve (AUC), 0.617; optimism-corrected AUC, 0.612) with a Brier score of 0.167. In the alternative model, TG18 grade III and a lower CALLY index remained independent predictors; however, predictive performance did not improve (AUC, 0.580; Brier score, 0.171). In the sensitivity analysis excluding operative time, severe DLC occurred in 378 patients (4.9%) and none of the candidate predictors remained independently associated with this endpoint. **Conclusions:** TG18 severity grade and preoperative CRP were independently associated with the composite DLC endpoint, which was predominantly driven by prolonged operative time. Discrimination was limited and no independent associations were identified for severe DLC when operative time was excluded. The alternative CALLY-based model showed lower discrimination than the conventional model. These findings do not support using the model to guide clinical decisions regarding severe operative difficulty.

## 1. Introduction

LC is the standard treatment for symptomatic gallbladder disease and remains one of the most commonly performed abdominal operations worldwide [1,2]. However, the procedure can be technically demanding in patients with AC, in whom inflammation, edema, adhesions, fibrosis and distorted biliary anatomy may compromise safe dissection [3,4]. This increased technical complexity is clinically important because DLC is associated with prolonged operative time, failure to achieve the critical view of safety, subtotal cholecystectomy, conversion to open surgery and increased perioperative morbidity [5,6]. Therefore, accurate preoperative risk assessment may improve operative planning, facilitate appropriate allocation of surgical expertise and perioperative resources and support more informed preoperative counseling regarding operative complexity and potential risks [7].

Numerous preoperative variables have been investigated as predictors of DLC [8]. The factors most consistently associated with operative difficulty include male sex, advanced age, obesity, recurrent biliary symptoms, previous upper abdominal surgery, prior endoscopic retrograde cholangiopancreatography, elevated inflammatory markers and ultrasonographic findings such as increased gallbladder wall thickness, pericholecystic fluid and impacted gallstones [2,9,10,11]. Nevertheless, the predictive performance of these variables has been inconsistent, with substantial variability in the strength and reproducibility of the reported associations across studies [2,8]. This heterogeneity likely reflects differences in patient populations, disease severity, definitions of operative difficulty and study methodology [12,13]. Consequently, reliable preoperative risk stratification remains challenging in routine clinical practice.

Although several studies have explored predictors of DLC, important limitations remain [2,9,14]. Many investigations have included relatively small cohorts, heterogeneous biliary populations or outcome definitions incorporating intraoperative grading systems, limiting their applicability to true preoperative risk assessment [8,12,14,15]. Moreover, studies focusing specifically on AC are relatively limited, despite the fact that these patients are particularly susceptible to operative difficulty because of active inflammation and distorted anatomy [3,11,16,17]. As a result, prediction models derived from broader biliary populations may not accurately reflect the preoperative risk profile of patients undergoing surgery for AC [18,19].

In this context, a large study focused exclusively on AC and based entirely on preoperative variables may provide clinically meaningful information for surgical risk stratification before surgery [18]. In addition to conventional clinical, laboratory and ultrasonographic parameters, composite immunonutritional markers may offer incremental predictive value by reflecting the combined effects of systemic inflammation, nutritional reserve and immune status [20]. Among these markers, the CALLY index is of particular interest because it integrates biologically relevant information on systemic inflammation, nutritional status and immune competence, all of which may contribute to operative difficulty in AC [3,21]. Accordingly, evaluating conventional preoperative predictors alongside a model incorporating the CALLY index may further refine preoperative risk stratification in this patient population [18,21].

Previous studies have demonstrated associations of TG18 severity grade and preoperative CRP with operative difficulty, including the predictive value of their combined assessment. Accordingly, the present study was not designed to propose TG18 grade or CRP as novel predictors. Instead, it aimed to confirm and extend the existing evidence by evaluating these variables in a substantially larger cohort restricted to AC and by comprehensively assessing model discrimination, calibration, clinical utility and internal validity. A secondary objective was to compare the predictive performance of the conventional model with that of an alternative model in which the CALLY index replaced its component variables.

## 2. Materials and Methods

### 2.1. Study Design and Setting

This retrospective single-center cohort study was conducted to develop and internally validate a preoperative prediction model for DLC in patients undergoing surgery for AC. The study was performed in the Department of General Surgery at Mersin City Training and Research Hospital, a tertiary referral center. The institution is a 1300-bed tertiary referral hospital with 52 operating rooms, seven of which are routinely allocated to the Department of General Surgery. The department comprises 19 surgeons and provides continuous emergency surgical services, including weekends. Consecutive patients treated between 1 January 2022 and 31 December 2025 were assessed for eligibility. This study was designed and reported in accordance with the Transparent Reporting of a Multivariable Prediction Model for Individual Prognosis or Diagnosis (TRIPOD) statement. The study protocol was approved by the Non-Interventional Clinical Research Ethics Committee of Mersin City Training and Research Hospital (date: 25 March 2026; number: 2026/161). The study was conducted in accordance with the ethical principles of the Declaration of Helsinki. Because of the retrospective design and use of anonymized data, the requirement for informed consent was waived by the Ethics Committee.

### 2.2. Study Population

Adult patients who underwent LC for AC during the study period were considered for inclusion. AC was diagnosed on the basis of clinical, laboratory and radiological findings in accordance with the principles of the TG18 and routine institutional practice. Eligible patients were identified from the electronically maintained operating-room registry within the hospital information system. Patients were excluded if surgery was performed for an indication other than AC, essential preoperative clinical, laboratory or imaging data were missing, primary open cholecystectomy was planned, concomitant major hepatobiliary or upper abdominal procedures were performed, or operative documentation was incomplete.

Potential duplicate entries were identified by cross-checking the unique patient identification number, date of surgery and operative record number. Duplicate records were excluded before formation of the final analytical cohort, as reported in Figure 1, ensuring that each surgical episode was included only once. Only patients with adequate perioperative documentation to determine the study outcomes were included in the final analysis.

### 2.3. Surgical Management, Data Sources, and Candidate Predictors

All patients included in the study were managed at the study institution and underwent LC according to routine institutional practice. Perioperative clinical data were retrospectively extracted from the institutional electronic medical record system, operative reports, anesthesia charts, laboratory records and radiology reports. All variables were reviewed for completeness, plausibility and internal consistency. Only variables available before surgery were considered for model development. The preoperative variables assessed included age, sex, body mass index (BMI), American Society of Anesthesiologists (ASA) physical status, TG18 severity grade, symptom duration, white blood cell count, CRP, albumin, lymphocyte count, total bilirubin, gallbladder wall thickness, pericholecystic fluid, impacted stone and gallbladder distension. Because the CALLY index reflects the combined inflammatory, nutritional and immune status of the patient, it was also evaluated as a composite predictor. However, because the CALLY index is mathematically derived from CRP, albumin and lymphocyte count, it was not entered together with these component variables in the primary conventional multivariable model.

The C-reactive protein–albumin–lymphocyte (CALLY) index was calculated using the following formula: CALLY index = [serum albumin (g/L) × absolute lymphocyte count (×10^9^/L)]/CRP (mg/L). Because serum albumin was recorded in g/dL in the institutional database, albumin values were multiplied by 10 to convert them to g/L before calculation. Thus, the equivalent formula applied to the study data was: [albumin (g/dL) × 10 × lymphocyte count (×10^9^/L)]/CRP (mg/L). The resulting value was reported as an index without a physical unit.

### 2.4. Outcome Definition

The primary outcome of the study was DLC. DLC was defined as the presence of at least one of the following intraoperative indicators of operative difficulty: operative time >90 min, conversion to open surgery, subtotal cholecystectomy or a Nassar operative difficulty grade ≥4. Operative time, conversion status and subtotal cholecystectomy were obtained from the operative records. The Nassar operative difficulty grade was retrospectively assigned by a single assessor based on the operative documentation recorded in the surgical notes. Only patients with sufficient operative documentation to classify the outcome were included in the analysis. As a sensitivity analysis, a more stringent definition of severe DLC was used, defined as conversion to open surgery, subtotal cholecystectomy or Nassar grade ≥4, thereby excluding operative time alone from the outcome definition.

The composite outcome was designed to capture complementary manifestations of operative difficulty rather than to imply that its individual components represented equivalent levels of severity. Prolonged operative time reflects increased technical complexity and resource utilization, whereas conversion to open surgery and subtotal cholecystectomy represent bailout strategies used when safe completion of conventional LC is not feasible. A Nassar grade ≥4 reflects severe intraoperative anatomical difficulty. Combining these components provided a pragmatic and multidimensional definition of DLC that incorporated procedural duration, intraoperative anatomy and the need for alternative operative strategies. Because conversion, subtotal cholecystectomy and Nassar grade ≥4 may represent more severe forms of difficulty than prolonged operative time alone, these outcomes were also evaluated separately as a more stringent composite endpoint in the sensitivity analysis.

### 2.5. Development of the Simplified Clinical Score

For exploratory simplification of the final prediction model, a simplified clinical score was derived from the regression coefficients of the final multivariable model. The relative contribution of each retained predictor was converted into an integer-based point value after scaling and rounding the regression coefficients and the total score was calculated for each patient. Patients were categorized into low-, intermediate- and high-risk groups using exploratory, data-derived score cutoffs selected from the score distribution in the present cohort, and the observed frequency of DLC was calculated within each category. These cutoffs were not prespecified before analysis.

### 2.6. Statistical Analysis

All statistical analyses were performed using IBM SPSS Statistics for Windows version 25.0 (IBM Corp., Armonk, NY, USA). Model performance plots, including receiver operating characteristic (ROC) curves, calibration plots and decision curve analyses, were generated using Python version 3.11.9 (Python Software Foundation, Wilmington, DE, USA). Continuous variables were evaluated for distributional characteristics and are presented as medians (interquartile range [IQR]), whereas categorical variables are presented as numbers (percentages). Baseline characteristics were compared between patients with and without DLC. Continuous variables were compared using the Mann–Whitney U test and categorical variables were compared using the chi-square test, as appropriate. Associations between preoperative predictors and DLC were first examined using univariable logistic regression analysis. Continuous predictors were analyzed on their original scales without arbitrary categorization and odds ratios (ORs) with 95% confidence intervals (CIs) were calculated for each predictor. Linearity in the logit was assessed using the Box–Tidwell approach. No significant departure from linearity was identified for age, BMI, symptom duration or gallbladder wall thickness; however, CRP showed evidence of nonlinearity. Therefore, a sensitivity analysis was performed using logarithmically transformed CRP.

A multivariable logistic regression model was constructed to identify independent preoperative predictors of DLC. The primary conventional model included prespecified clinically relevant preoperative variables: TG18 grade, age, sex, BMI, symptom duration, CRP, albumin, total bilirubin, gallbladder wall thickness, pericholecystic fluid, impacted stone and distended gallbladder. To preserve model parsimony and avoid redundancy, ASA physical status and white blood cell count were examined in descriptive and univariable analyses but were not forced into the primary multivariable model. Lymphocyte count was not co-entered into the conventional model because the inflammatory-nutritional construct was examined separately through its individual components and through the alternative CALLY-based model. No automatic stepwise variable selection procedure was used. To address collinearity, the CALLY index was not entered into the conventional model. Instead, an alternative multivariable model was constructed in which the CALLY index replaced its component variables.

Model performance was evaluated in terms of discrimination, calibration and clinical utility. Discrimination was assessed by calculating the AUC. Calibration was assessed using calibration plots and the Brier score. The diagnostic performance of the final multivariable model and simplified clinical score was described using data-derived classification thresholds. The predicted-probability threshold of 0.316 was selected by maximizing the Youden index in the development cohort. The score threshold of ≥9 corresponded to the exploratory high-risk category derived from the score distribution in the present dataset. Neither threshold was prespecified before analysis. In an additional exploratory analysis, the intermediate-risk and high-risk categories were combined into a non-low-risk group to further evaluate the ability of the simplified clinical score to identify a lower-risk subgroup. Clinical utility was assessed using decision curve analysis across clinically relevant threshold probabilities. Decision curve analysis was based on apparent predictions from the development cohort; no optimism correction was applied to the estimated net benefit. Internal validation of the final model was performed using bootstrap resampling with 1000 repetitions. Apparent model performance was first assessed in the full analytical dataset. Bootstrap-based internal validation was then used to estimate optimism and assess the stability of model discrimination and calibration. Patients with missing essential preoperative variables were excluded before analysis; therefore, all regression analyses were performed using a complete-case dataset. As a sensitivity analysis, regression analyses were repeated using a more stringent definition of severe DLC. All tests were two-sided, and a *p* value < 0.05 was considered statistically significant.

## 3. Results

A total of 8530 patients were assessed for eligibility during the study period. After exclusion of 794 patients according to the predefined criteria, 7736 patients were included in the final analysis (Figure 1). Of these, 1736 (22.4%) met the study definition of DLC, whereas 6000 (77.6%) did not.

Among the 1736 patients who met the composite DLC definition, 1432 (82.5%) fulfilled the operative-time criterion, 117 (6.7%) underwent conversion to open surgery, 106 (6.1%) underwent subtotal cholecystectomy and 177 (10.2%) had a Nassar grade ≥4. Because individual patients could meet more than one criterion, these frequencies were not mutually exclusive. Overall, 1646 patients (94.8%) met one component, 84 (4.8%) met two components and six (0.3%) met three components; no patient met all four components. Operative time alone accounted for 1358 DLC cases (78.2%). The detailed overlap between the individual components is presented in Supplementary Table S1.

Bile duct injury occurred in 30 patients (0.4%). According to the Clavien–Dindo classification, 5000 patients (64.6%) had no postoperative complication, while 1500 (19.4%), 800 (10.3%), 250 (3.2%), 120 (1.6%), 50 (0.6%) and 16 (0.2%) experienced grade I, II, IIIa, IIIb, IV and V morbidity, respectively. Overall, 436 patients (5.6%) experienced Clavien–Dindo grade ≥ IIIa morbidity. The distribution of bile duct injury and postoperative morbidity according to DLC status is presented in Supplementary Table S2.

The baseline characteristics according to DLC status are summarized in Table 1. Compared with patients without DLC, those with DLC had higher median preoperative CRP levels (147.1 vs. 115.3 mg/L; *p* < 0.001), lower median CALLY index values (0.60 vs. 0.79; *p* < 0.001) and a higher frequency of impacted stones (32.8% vs. 30.0%; *p* = 0.030). The distribution of TG18 grades also differed significantly between the groups (*p* = 0.022), with a lower proportion of Grade I disease and higher proportions of Grades II and III disease in the DLC group (Table 1).

In univariable logistic regression analysis (Table 2), TG18 grade II (OR, 1.125; 95% CI, 1.002–1.262; *p* = 0.046), TG18 grade III (OR, 1.239; 95% CI, 1.037–1.480; *p* = 0.018), CRP (OR, 1.006 per mg/L; 95% CI, 1.005–1.007; *p* < 0.001), impacted stone (OR, 1.137; 95% CI, 1.014–1.275; *p* = 0.028) and the CALLY index (OR, 0.864; 95% CI, 0.832–0.896; *p* < 0.001) were significantly associated with DLC.

In the multivariable logistic regression model (Table 3), TG18 grade II (adjusted OR, 1.151; 95% CI, 1.008–1.315; *p* = 0.038), TG18 grade III (adjusted OR, 1.306; 95% CI, 1.044–1.634; *p* = 0.019) and CRP (adjusted OR, 1.006 per mg/L; 95% CI, 1.005–1.007; *p* < 0.001) remained independently associated with DLC. Impacted stone and the remaining covariates were not independently associated with DLC after adjustment.

The observed DLC rates across the simplified clinical score categories are shown in Table 4. The DLC rates were 13.0% (14/108) in the low-risk group, 20.4% (343/1680) in the intermediate-risk group and 23.2% (1379/5948) in the high-risk group. The corresponding proportions of patients without DLC were 87.0%, 79.6% and 76.8%, respectively. When the intermediate-risk and high-risk groups were combined, the observed DLC rate was 22.6% (1722/7628) in the non-low-risk group, compared with 13.0% (14/108) in the low-risk group.

The predictive performances of the final multivariable model and the simplified clinical score are summarized in Table 5. The final multivariable model achieved an area under the receiver operating characteristic curve (AUC) of 0.617, whereas the simplified clinical score achieved an AUC of 0.525 (Figure 2). The Brier score of the final multivariable model was 0.167. Using the data-derived classification thresholds, the final model yielded a specificity of 89.4%, a positive predictive value of 44.5%, a negative predictive value of 81.4%, an accuracy of 75.9%, and a sensitivity of 29.4%. The corresponding values for the simplified clinical score were 23.8%, 23.2%, 80.0%, 36.3%, and 79.4%, respectively. The calibration plot of the final multivariable model is shown in Figure 3. Deviation from the ideal calibration line was observed at higher predicted probabilities. Decision curve analysis is presented in Figure 4. The final multivariable model showed a higher net benefit than the simplified clinical score across intermediate threshold probabilities. Internal validation using 1000 bootstrap resamples is summarized in Supplementary Table S3. The apparent AUC decreased from 0.617 to 0.612 after optimism correction, whereas the Brier score changed from 0.167 to 0.168.

A sensitivity analysis using a more stringent definition of severe DLC, defined as conversion to open surgery, subtotal cholecystectomy, or Nassar grade ≥4, is presented in Supplementary Table S4. In this analysis, no candidate variable remained independently associated with the outcome, although a distended gallbladder showed borderline significance (adjusted OR, 1.109; 95% CI, 0.984–1.250; *p* = 0.089).

An alternative multivariable model incorporating the CALLY index instead of its component variables is presented in Supplementary Table S5. In this model, TG18 grade III (adjusted OR, 1.266; 95% CI, 1.015–1.580; *p* = 0.037) and the CALLY index (adjusted OR, 0.864; 95% CI, 0.833–0.897; *p* < 0.001) remained independently associated with DLC, whereas TG18 grade II showed borderline significance (adjusted OR, 1.131; 95% CI, 0.991–1.291; *p* = 0.068). The alternative CALLY-based model yielded an AUC of 0.580 and a Brier score of 0.171. 

## 4. Discussion

In this retrospective single-center cohort study of 7736 patients who underwent LC for AC, TG18 grades II and III and higher preoperative CRP levels remained independently associated with DLC in the primary multivariable model. In the alternative model, TG18 grade III and a lower CALLY index remained independently associated with DLC, whereas TG18 grade II showed borderline significance. Overall, these findings indicate that clinically relevant preoperative risk factors can be identified before surgery using routinely available clinical and laboratory parameters, although the overall discriminatory performance of the model remained modest.

The observed associations between TG18 severity grade and CRP are biologically and clinically plausible. TG18 grading reflects the severity of AC and therefore captures the extent of local inflammation, tissue edema, distorted biliary anatomy and technical complexity encountered during surgery [22,23,24]. Similarly, CRP is an objective and readily available biomarker of systemic inflammation that correlates with the intensity of the underlying inflammatory process [22,23,25]. The independent associations of TG18 grade and CRP observed in the present study therefore reinforce the role of inflammatory severity as a key preoperative determinant of DLC and suggest that these variables provide complementary information for preoperative risk assessment [22,26]. These associations should be interpreted as confirmation and extension of existing evidence rather than the identification of novel predictors. In particular, Mishima et al. previously demonstrated that combining preoperative CRP with TG18 grading could improve the prediction of surgical difficulty in early LC [22]. The present study extends this work by evaluating these variables in a substantially larger cohort and by assessing model performance beyond statistical associations through calibration, Brier score, decision curve analysis and bootstrap internal validation. Nevertheless, the modest optimism-corrected AUC indicates that the increased sample size and broader performance assessment did not translate into strong individual-level discrimination.

The present findings should also be interpreted in the context of the existing literature. Previous studies have identified a wide range of factors associated with DLC, including male sex, older age, previous abdominal surgery, gallbladder wall thickness, a history of endoscopic retrograde cholangiopancreatography (ERCP) and inflammatory markers [27,28,29,30,31]. In contrast, several of these variables were not independently associated with DLC after multivariable adjustment in the present study. This difference may reflect variations in study populations, predictor selection and outcome definitions across previous investigations. Because our analysis was restricted to patients with AC and exclusively included variables available before surgery, markers reflecting the severity of the current inflammatory process, particularly TG18 grade and CRP, may have greater prognostic relevance than broader demographic or imaging-derived characteristics [23,32,33,34]. A similar pattern was observed for impacted stones. Although impacted stones were more common in patients with DLC and were associated with the outcome in the univariable analysis, this association was no longer significant after multivariable adjustment. One possible explanation is that their apparent effect is largely mediated through the overall inflammatory severity already captured by TG18 grade and CRP, rather than representing an independent predictor.

Another important finding of the present study was the separate evaluation of the CALLY index. Because the CALLY index is derived from CRP, albumin and lymphocyte count, it was assessed in an alternative multivariable model to avoid collinearity with its component variables [33,34,35]. In this model, a lower CALLY index remained independently associated with DLC, suggesting that combined inflammatory, nutritional and immune status may represent a biologically relevant dimension of operative vulnerability. This finding is not unexpected because the inflammatory information captured by the CALLY index substantially overlaps with that provided by CRP alone. However, the CALLY-based model did not outperform the conventional multivariable model, indicating that the observed association of CALLY with DLC did not translate into superior predictive performance of the alternative model. Consistent with this interpretation, the CALLY-based model showed lower discrimination (AUC, 0.580) and a slightly higher Brier score (0.171) than the conventional model. The comparative performance of the alternative CALLY-based model is informative, as it indicates that using CALLY in place of CRP and albumin, with lymphocyte count not entered separately in either model, does not improve preoperative prediction in this setting. This negative finding may help avoid unnecessary complexity in future prediction models; however, the comparison evaluates alternative model specifications and does not establish the incremental predictive value of adding CALLY to the conventional model.

Exploratory simplification of the multivariable model resulted in a substantial loss of discrimination with an AUC of 0.525 compared with 0.617 for the full model. Although observed DLC rates increased across the lower-risk, intermediate-risk and higher-risk categories (13.0%, 20.4%, and 23.2%, respectively), the separation between the latter two categories was small. Moreover, the lower-risk category included only 108 patients and the category boundaries were derived from the same cohort. These descriptive differences therefore do not establish clinically useful risk stratification. The simplified risk is reported to document the performance consequences of model simplification, rather than to recommend its use for individual prediction or clinical decision-making. Bootstrap validation of the full model showed little optimism with an AUC of 0.612 after correction, but this validation does not extend to the simplified risk.

These performance metrics should be interpreted within the clinical context of AC. DLC is a multifactorial and partly operator-dependent outcome influenced not only by preoperative disease severity but also by intraoperative anatomy, technical factors and surgeon-related variables that cannot be fully assessed before surgery [2,36,37,38]. Consequently, prediction models developed exclusively from preoperative variables are inherently limited because several important determinants of operative difficulty become apparent only during surgery. The observed sensitivity of 29.4% applies specifically to the data-derived probability threshold of 0.316; alternative thresholds would involve different sensitivity–specificity trade-offs, but their clinical usefulness has not been established. Decision curve analysis demonstrated a modest net benefit across clinically relevant intermediate threshold probabilities. Accordingly, the present model should be considered an exploratory approach to broad preoperative risk stratification rather than a stand-alone bedside tool or a definitive predictor of operative difficulty.

The primary composite outcome was predominantly driven by prolonged operative time with 1358 of the 1736 DLC cases (78.2%) meeting this criterion alone. This predominance does not necessarily imply an absence of clinically meaningful technical difficulty. Inflammation, adhesions, distorted anatomy and the need for meticulous dissection may prolong surgery even when the procedure is completed laparoscopically without conversion or subtotal cholecystectomy. Accordingly, operative duration was included to capture a broader spectrum of operative difficulty beyond severe anatomical findings and bailout procedures. However, the specific reasons for prolonged surgery could not be established retrospectively and operative time may also be influenced by surgeon-related and institutional factors. When operative time was excluded, the severe-DLC endpoint, comprising conversion, subtotal cholecystectomy or Nassar grade ≥4, occurred in 378 patients (4.9%) and none of the candidate variables remained independently associated with this outcome. The primary findings should therefore be interpreted as associations with a composite endpoint predominantly driven by prolonged operative duration; they do not establish reliable prediction of its more severe components.

The present study has several strengths. It included a large consecutive cohort of 7736 patients and focused exclusively on AC while restricting model development to variables available before surgery, thereby preserving direct preoperative applicability. To our knowledge, this represents one of the largest cohorts specifically evaluating preoperative predictors of DLC in patients with AC. The contribution of the study lies primarily in the large-scale evaluation, internal validation and comprehensive performance assessment of previously identified predictors, together with the direct comparison of the conventional and alternative CALLY-based models. Collinearity was addressed transparently by evaluating separate conventional and CALLY-based models and model performance was comprehensively assessed using discrimination, calibration, decision curve analysis, sensitivity analyses and bootstrap internal validation within a TRIPOD-oriented framework. 

Several limitations should be acknowledged. First, the retrospective single-center design may have introduced selection and information bias and may limit the generalizability of the findings. Second, complete-case analysis may have introduced bias related to missing data; because the screening log retained only the overall number of patients excluded for missing essential data, variable-specific missingness patterns could not be reconstructed and multiple imputation was not feasible. Third, important determinants of operative difficulty, particularly surgeon-related factors, temporal influences and detailed radiologic features, could not be fully captured. Because the study was conducted at a high-volume tertiary referral center, the findings may not be directly generalizable to lower-volume institutions or different healthcare settings. Nassar grades were retrospectively assigned from operative notes by a single assessor, making their accuracy dependent on the completeness and detail of the documentation. This may have introduced outcome misclassification, and interobserver reproducibility was not assessed. In addition, the primary composite outcome was predominantly driven by prolonged operative time. When operative time was excluded and a more stringent severe-DLC definition comprising conversion, subtotal cholecystectomy or Nassar grade ≥4 was applied, no candidate variable remained independently associated with the outcome. Therefore, the primary findings appear to relate mainly to prolonged operative duration and broader technical difficulty rather than to severe anatomical difficulty or the need for bailout procedures. The model should consequently not be used to predict conversion or subtotal cholecystectomy. Furthermore, the decision-curve analysis was based on apparent predictions without optimism correction and may therefore overestimate the model’s clinical net benefit. Finally, although bootstrap validation demonstrated stable internal model performance, external multicenter validation is required before routine clinical implementation.

## 5. Conclusions

In this large retrospective cohort of patients undergoing LC for AC, TG18 severity grade and preoperative CRP were independently associated with the composite DLC endpoint, predominantly driven by prolonged operative duration. However, model discrimination remained limited after internal validation. When operative time was excluded, none of the candidate predictors remained independently associated with severe DLC, defined as conversion to open surgery, subtotal cholecystectomy, or Nassar grade ≥4. The alternative CALLY-based model showed lower discrimination than the conventional model. These results highlight the distinction between associations with a broad composite endpoint and prediction of its more severe components. The present findings do not establish sufficient predictive performance to support clinical decisions regarding severe operative difficulty; further model development and external validation are required.

## Figures and Tables

**Figure 1:**
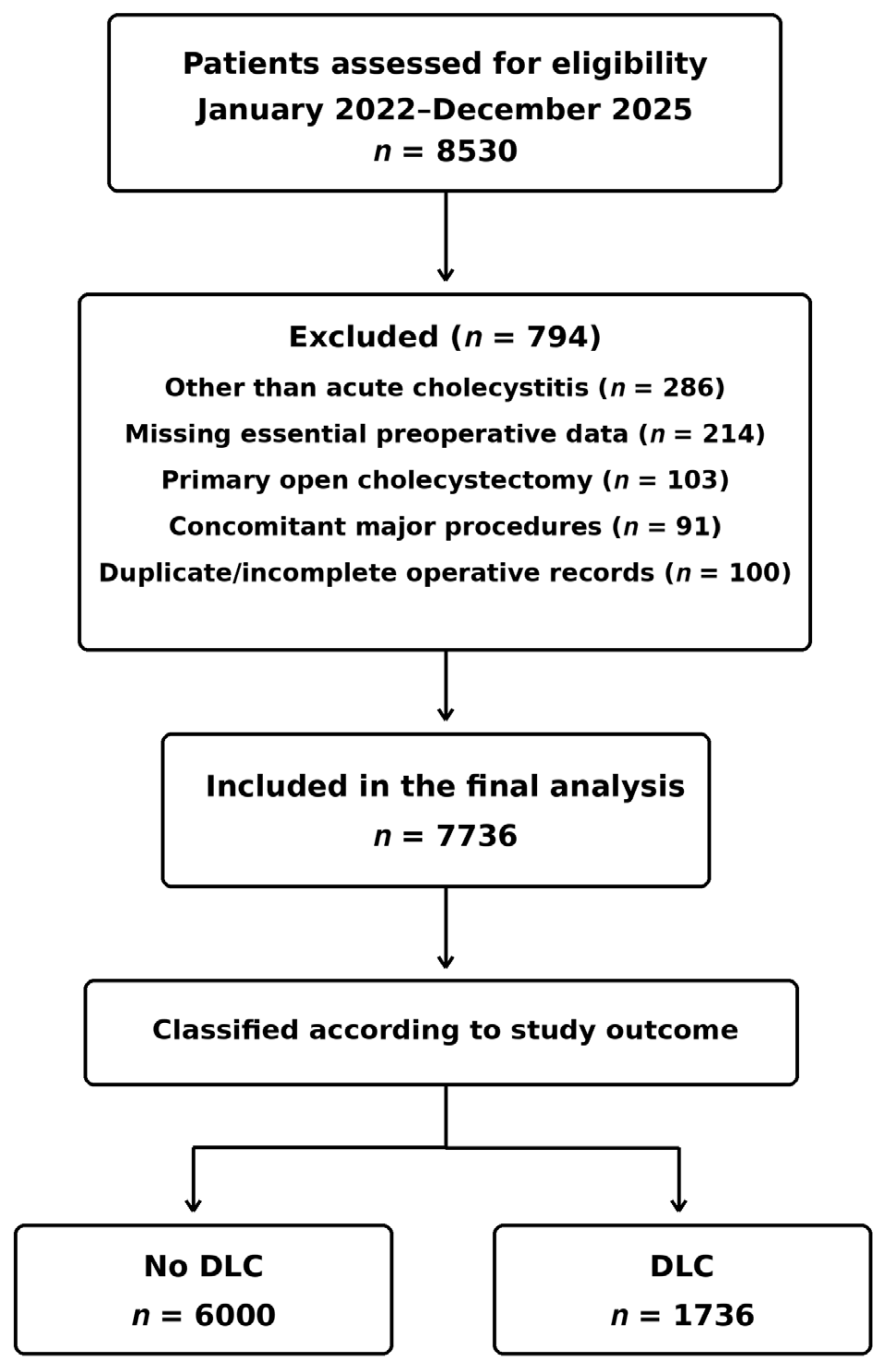
Flow diagram of patient selection. Of 8530 patients assessed for eligibility, 794 were excluded and 7736 were included in the final analysis. DLC, difficult laparoscopic cholecystectomy.

**Figure 2:**
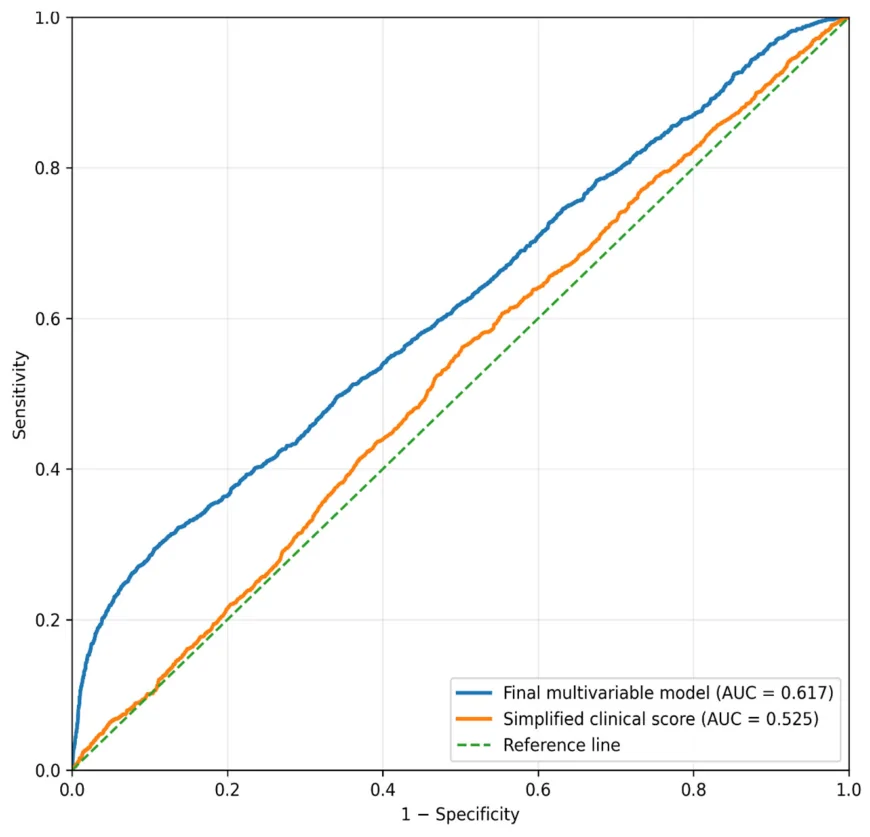
Receiver operating characteristic curves of the final multivariable model and the simplified clinical score for predicting DLC. The final multivariable model showed better discrimination than the simplified clinical score (AUC, 0.617 vs. 0.525).

**Figure 3:**
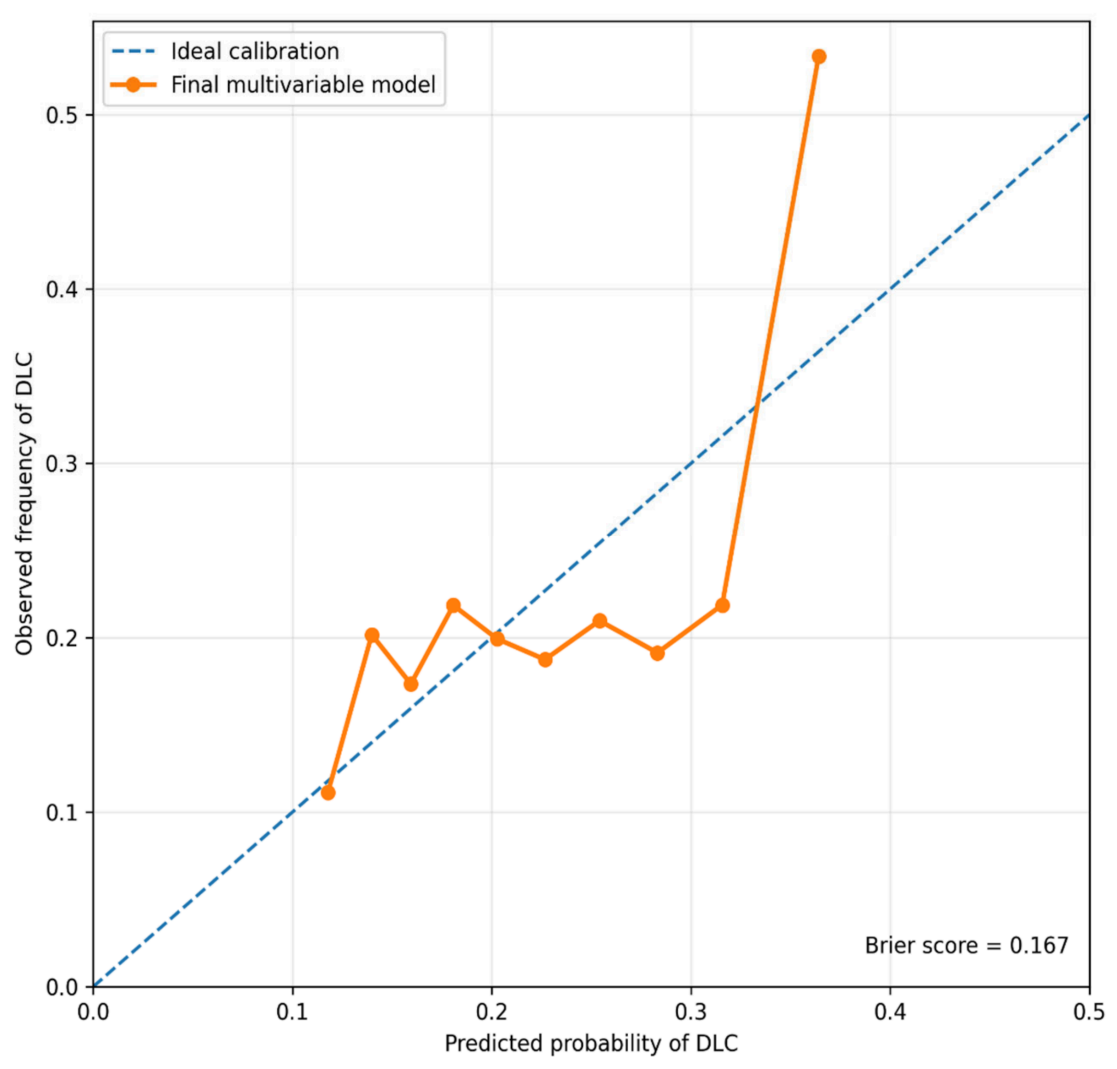
Calibration plot of the final multivariable model for predicting DLC. The dashed diagonal line represents ideal calibration, and the plotted line with markers represents the observed proportions of DLC across deciles of predicted risk. The model showed reasonable agreement in the lower-to-intermediate range of predicted probabilities, with greater deviation at higher predicted probabilities. The Brier score was 0.167.

**Figure 4:**
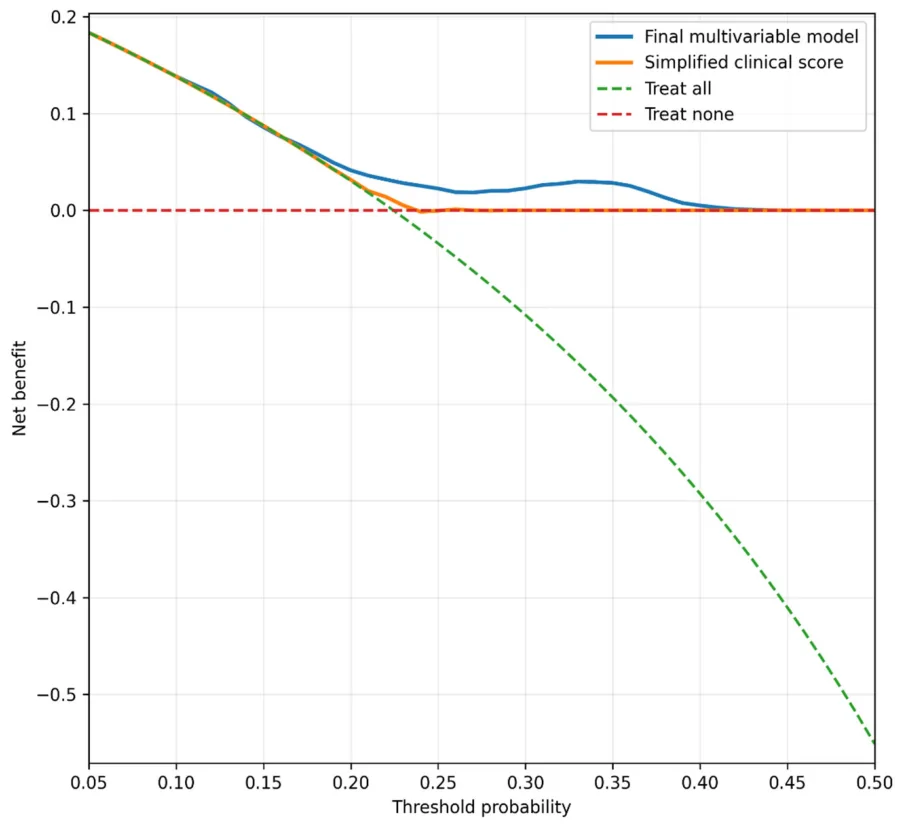
Decision curve analysis of the final multivariable model and simplified clinical score for predicting DLC. Across an intermediate range of threshold probabilities, the final multivariable model showed greater net benefit than the simplified clinical score and the default treat-all and treat-none strategies. The simplified clinical score provided minimal additional clinical utility across most threshold probabilities. The decision-curve analysis was based on apparent predictions from the development cohort and was not corrected for optimism. Therefore, the estimated net benefit should be interpreted as exploratory and potentially optimistic.

**Table 1:** Baseline characteristics of the study population according to DLC status.

Variable	Overall (*n* = 7736)	No DLC (*n* = 6000)	DLC (*n* = 1736)	*p* Value
Age, years	55.0 (43.0–67.0)	55.0 (43.0–67.0)	55.0 (43.0–67.0)	0.363
BMI, kg/m^2^	29.9 (23.9–35.9)	29.9 (23.8–35.9)	30.2 (24.2–35.8)	0.528
Symptom duration, h	67.4 (43.2–100.2)	67.1 (43.0–99.8)	69.3 (43.9–101.3)	0.153
WBC, ×10^9^/L	10.89 (7.48–14.43)	10.88 (7.49–14.42)	10.95 (7.45–14.51)	0.782
CRP, mg/L	121.4 (62.9–183.4)	115.3 (58.2–174.8)	147.1 (81.3–218.6)	<0.001
Albumin, g/dL	3.66 (3.07–4.22)	3.66 (3.08–4.22)	3.67 (3.06–4.22)	0.8
Lymphocyte, ×10^9^/L	2.49 (1.49–3.50)	2.50 (1.48–3.51)	2.45 (1.50–3.49)	0.742
Total bilirubin, mg/dL	1.85 (1.03–2.67)	1.83 (1.01–2.67)	1.89 (1.08–2.69)	0.136
Gallbladder wall thickness, mm	6.60 (4.80–8.50)	6.60 (4.80–8.50)	6.60 (4.90–8.40)	0.531
CALLY index	0.73 (0.41–1.47)	0.79 (0.43–1.56)	0.60 (0.36–1.15)	<0.001
Male sex, *n* (%)	2036 (26.3)	1580 (26.3)	456 (26.3)	0.981
Pericholecystic fluid, *n* (%)	2248 (29.1)	1741 (29.0)	507 (29.2)	0.903
Impacted stone, *n* (%)	2370 (30.6)	1801 (30.0)	569 (32.8)	0.03
Distended gallbladder, *n* (%)	3235 (41.8)	2495 (41.6)	740 (42.6)	0.454
ASA III–IV, *n* (%)	3917 (50.6)	3021 (50.4)	896 (51.6)	0.368
TG18 grade, *n* (%)				0.022
Grade I	4254 (55.0)	3346 (55.8)	908 (52.3)	
Grade II	2707 (35.0)	2074 (34.6)	633 (36.5)	
Grade III	775 (10.0)	580 (9.7)	195 (11.2)	

Continuous variables are presented as median (interquartile range) and categorical variables as number (percentage). *p* values were calculated using the Mann–Whitney U test for continuous variables and the chi-square test for categorical variables.

**Table 2:** Univariable logistic regression analysis for predictors of DLC.

Variable	Univariable OR	95% CI	*p* Value
Age (per year)	1.001	0.998–1.004	0.419
Male sex	0.997	0.883–1.125	0.956
BMI (per kg/m^2^)	1.003	0.995–1.010	0.524
ASA II vs. I	1.031	0.885–1.202	0.693
ASA III vs. I	1.066	0.916–1.240	0.41
ASA IV vs. I	1.071	0.920–1.248	0.376
TG18 grade II vs. I	1.125	1.002–1.262	0.046
TG18 grade III vs. I	1.239	1.037–1.480	0.018
Symptom duration (per hour)	1.001	0.999–1.002	0.235
WBC (per 1 × 10^9^/L)	1.002	0.989–1.015	0.786
CRP (per mg/L)	1.006	1.005–1.007	<0.001
Albumin (per g/dL)	1.011	0.932–1.096	0.799
Lymphocyte (per 1 × 10^9^/L)	0.992	0.947–1.039	0.738
Total bilirubin (per mg/dL)	1.043	0.986–1.104	0.141
Gallbladder wall thickness (per mm)	1.005	0.988–1.022	0.576
Pericholecystic fluid	1.009	0.897–1.135	0.879
Impacted stone	1.137	1.014–1.275	0.028
Distended gallbladder	1.044	0.937–1.163	0.438
CALLY index	0.864	0.832–0.896	<0.001

Univariable logistic regression analysis was performed with DLC as the dependent variable. ORs are presented with 95% CIs.

**Table 3:** Multivariable logistic regression analysis for predictors of DLC.

Variable	Adjusted OR	95% CI	*p* Value
TG18 grade II vs. I	1.151	1.008–1.315	0.038
TG18 grade III vs. I	1.306	1.044–1.634	0.019
Age (per year)	1.001	0.998–1.004	0.425
Male sex	1.004	0.887–1.136	0.949
BMI (per kg/m^2^)	1.003	0.995–1.011	0.489
Symptom duration (per hour)	1.001	0.999–1.002	0.297
CRP (per mg/L)	1.006	1.005–1.007	<0.001
Albumin (per g/dL)	1.017	0.936–1.104	0.695
Total bilirubin (per mg/dL)	1.040	0.982–1.101	0.185
Gallbladder wall thickness (per mm)	1.002	0.985–1.019	0.853
Pericholecystic fluid	0.884	0.768–1.017	0.084
Impacted stone	1.091	0.964–1.234	0.168
Distended gallbladder	0.96	0.851–1.083	0.508

Multivariable logistic regression analysis was performed with DLC as the dependent variable. Adjusted ORs are presented with 95% CIs. The CALLY index was not entered into the multivariable model because of collinearity with its component variables. In the sensitivity analysis using logarithmically transformed CRP, log-CRP remained independently associated with DLC (adjusted OR, 1.597; 95% CI, 1.483–1.719; *p* < 0.001). Model discrimination remained modest (AUC, 0.611; Brier score, 0.169), supporting the overall interpretation of the primary analysis.

**Table 4:** Observed DLC rates according to simplified clinical risk categories.

Risk Category	Score Range	Total *n*	DLC, *n* (%)	No DLC, *n* (%)
Low	≤4.0	108	14 (13.0)	94 (87.0)
Intermediate	>4.0 to <9.0	1680	343 (20.4)	1337 (79.6)
High	≥9.0	5948	1379 (23.2)	4569 (76.8)

Risk categories were defined according to the total clinical score derived from the simplified scoring system. DLC was defined according to the study outcome variable.

**Table 5:** Performance of the final multivariable model and simplified clinical score for predicting DLC.

Metric	Final Multivariable Model	Simplified Clinical Score
Discrimination and calibration		
AUC	0.617	0.525
Brier score	0.167	—
Classification performance		
Classification threshold	Predicted probability ≥ 0.316	Score ≥ 9.0
Sensitivity (%)	29.4	79.4
Specificity (%)	89.4	23.8
Positive predictive value (%)	44.5	23.2
Negative predictive value (%)	81.4	80.0
Accuracy (%)	75.9	36.3

The final multivariable model included TG18 grade, age, sex, body mass index, symptom duration, C-reactive protein, albumin, total bilirubin, gallbladder wall thickness, pericholecystic fluid, impacted stone, and distended gallbladder. Diagnostic indices for the simplified clinical score were calculated using the exploratory, data-derived high-risk threshold of score ≥9.0.

## Data Availability

The datasets generated and/or analyzed during the current study are available from the corresponding author upon reasonable request.

## Supplementary Materials

The following supporting information can be downloaded at: https://www.mdpi.com/article/10.3390/diagnostics16193191/s1: Table S1: Frequency and Overlap of the Components Defining Difficult Laparoscopic Cholecystectomy; Table S2: Bile Duct Injury and Postoperative Morbidity According to DLC Status; Table S3: Bootstrap Internal Validation of the Final Multivariable Model for Predicting Difficult Laparoscopic Cholecystectomy; Table S4: Sensitivity Analysis Using Severe Difficult Laparoscopic Cholecystectomy as the Outcome; Table S5: Alternative Multivariable Logistic Regression Model Including the CALLY Index for Prediction of Difficult Laparoscopic Cholecystectomy.

## Abbreviations

The following abbreviations are used in this manuscript:

AC	acute cholecystitis
ASA	American Society of Anesthesiologists
AUC	area under the curve
BMI	body mass index
CALLY	C-reactive protein–albumin–lymphocyte
CI	confidence interval
CRP	C-reactive protein
DLC	difficult laparoscopic cholecystectomy
IQR	interquartile range
LC	laparoscopic cholecystectomy
OR	odds ratio
ROC	receiver operating characteristic
TG18	Tokyo Guidelines 2018
TRIPOD	Transparent Reporting of a Multivariable Prediction Model for Individual Prognosis or Diagnosis
