# Supplementary material for: Development and Internal Validation of a Preoperative Risk Stratification Model for Difficult Laparoscopic Cholecystectomy in Acute Cholecystitis
Source: Diagnostics (Basel). 2026 Oct 1;16(19):3191. doi: 10.3390/diagnostics16193191 (PMC13664390; doi:10.3390/diagnostics16193191)
Supplement: Supplementary file 1 [file diagnostics-16-03191-s001.zip › diagnostics-4493400-supplementary.pdf]

Supplementary Table S1. Frequency and overlap of the components defining difficult laparoscopic cholecystectomy

Exact combination	n	% of DLC
Operative time criterion only	1,358	78.2
Nassar grade ≥4 only	132	7.6
Subtotal cholecystectomy only	81	4.7
Conversion only	75	4.3
Operative time + Nassar grade ≥4	30	1.7
Operative time + conversion	28	1.6
Operative time + subtotal cholecystectomy	12	0.7
Subtotal cholecystectomy + Nassar grade ≥4	6	0.3
Conversion + Nassar grade ≥4	4	0.2
Conversion + subtotal cholecystectomy	4	0.2
Operative time + conversion + Nassar grade ≥4	3	0.2
Conversion + subtotal cholecystectomy + Nassar grade ≥4	2	0.1
Operative time + conversion + subtotal cholecystectomy	1	0.1
**Total DLC**	**1,736**	**100.0**

DLC, difficult laparoscopic cholecystectomy. Percentages were calculated using the 1,736 patients with DLC as the denominator. The operative-time criterion was applied consistently with the study outcome coding. Combinations are mutually exclusive.

Supplementary Table S2. Bile Duct Injury and Postoperative Morbidity According to DLC Status

Outcome	Overall (n=7,736)	No DLC (n=6,000)	DLC (n=1,736)	p value
Bile duct injury	30 (0.4)	27 (0.4)	3 (0.2)	0.156
Clavien–Dindo grade				0.745
0 (no complication)	5,000 (64.6)	3,878 (64.6)	1,122 (64.6)	
I	1,500 (19.4)	1,164 (19.4)	336 (19.4)	
II	800 (10.3)	624 (10.4)	176 (10.1)	
IIIa	250 (3.2)	187 (3.1)	63 (3.6)	
IIIb	120 (1.6)	91 (1.5)	29 (1.7)	
IV	50 (0.6)	42 (0.7)	8 (0.5)	
V	16 (0.2)	14 (0.2)	2 (0.1)	
Clavien–Dindo grade ≥IIIa	436 (5.6)	334 (5.6)	102 (5.9)	0.660

Data are presented as n (%). DLC, difficult laparoscopic cholecystectomy. Bile duct injury was reported separately as a specific operative complication. Postoperative morbidity was classified according to the Clavien–Dindo classification. Comparisons were performed using the chi-square test.

Supplementary Table S3. Bootstrap internal validation of the final multivariable model for predicting difficult laparoscopic cholecystectomy

**Performance metric**	**Apparent performance**	**Optimism**	**Optimism-corrected performance**
Area under the receiver operating characteristic curve (AUC)	0.617	0.005	0.612
Brier score	0.167	-0.001	0.168
**Additional model stability index**			
**Metric**	**Value**		
Apparent calibration slope	1.000		
Number of bootstrap resamples	1,000		

AUC, area under the receiver operating characteristic curve. Internal validation was performed using 1,000 bootstrap resamples. Optimism-corrected performance was estimated by subtracting the bootstrap-derived optimism from the apparent model performance. Lower Brier scores indicate better overall predictive accuracy.

Supplementary Table S4. Sensitivity analysis using severe difficult laparoscopic cholecystectomy as the outcome

Definition of severe DLC: conversion to open surgery, subtotal cholecystectomy, or Nassar grade ≥4.

Variable	Univariable OR	95% CI	p value	Adjusted OR	95% CI	p value
TG18 grade II vs I	1.033	0.920–1.161	0.58	0.981	0.858–1.120	0.772
TG18 grade III vs I	1.012	0.841–1.218	0.899	0.911	0.726–1.143	0.42
Age (per year)	0.999	0.996–1.002	0.688	0.999	0.996–1.002	0.67
Male sex	0.927	0.819–1.049	0.229	0.924	0.816–1.046	0.209
BMI (per kg/m²)	1.002	0.994–1.010	0.645	1.002	0.994–1.010	0.639
Symptom duration (per hour)	1.000	0.999–1.002	0.857	1.000	0.999–1.002	0.855
CRP (per mg/L)	1.000	0.999–1.000	0.356	1.000	0.999–1.000	0.328
Albumin (per g/dL)	1.025	0.945–1.113	0.55	1.021	0.941–1.108	0.618
Total bilirubin (per mg/dL)	0.969	0.915–1.025	0.272	0.97	0.916–1.026	0.288
GB wall thickness (per mm)	1.008	0.991–1.025	0.379	1.009	0.992–1.026	0.307
Pericholecystic fluid	1.089	0.968–1.225	0.155	1.108	0.965–1.271	0.146
Impacted stone	0.957	0.851–1.077	0.469	0.937	0.828–1.062	0.308
Distended gallbladder	1.098	0.985–1.224	0.093	1.109	0.984–1.250	0.089

Severe difficult laparoscopic cholecystectomy was defined as conversion to open surgery, subtotal cholecystectomy, or Nassar grade ≥4. Adjusted odds ratios were obtained from a multivariable logistic regression model including TG18 grade, age, sex, BMI, symptom duration, CRP, albumin, total bilirubin, gallbladder wall thickness, pericholecystic fluid, impacted stone, and distended gallbladder.

Supplementary Table S5. Alternative multivariable logistic regression model including the CALLY index for prediction of difficult laparoscopic cholecystectomy

A. Multivariable logistic regression results

Variable	Adjusted OR	95% CI	p value
TG18 grade II vs I	1.131	0.991–1.291	0.068
TG18 grade III vs I	1.266	1.015–1.580	0.037
Age (per year)	1.001	0.998–1.004	0.52
Male sex	1.002	0.886–1.132	0.981
BMI (per kg/m²)	1.003	0.995–1.011	0.43
Symptom duration (per hour)	1.001	0.999–1.002	0.259
CALLY index	0.864	0.833–0.897	<0.001
Total bilirubin (per mg/dL)	1.038	0.980–1.099	0.203
Gallbladder wall thickness (per mm)	1.002	0.985–1.019	0.789
Pericholecystic fluid	0.895	0.779–1.029	0.117
Impacted stone	1.085	0.960–1.225	0.193
Distended gallbladder	0.969	0.859–1.092	0.602

B. Model performance

Metric	Alternative CALLY-based model
Area under the receiver operating characteristic curve (AUC)	0.58
Brier score	0.171

Adjusted ORs were obtained from an alternative multivariable logistic regression model including TG18 grade, age, sex, BMI, symptom duration, CALLY index, total bilirubin, gallbladder wall thickness, pericholecystic fluid, impacted stone, and distended gallbladder. The model was constructed to avoid collinearity between CALLY and its component variables. Model performance is shown for comparison with the conventional multivariable model. Lower Brier scores indicate better overall predictive accuracy. OR, odds ratio; CI, confidence interval; BMI, body mass index; TG18, Tokyo Guidelines 2018; CALLY, C-reactive protein–albumin–lymphocyte index; AUC, area under the receiver operating characteristic curve.
